# Validation of entrustable professional activities for use in neonatal care residency programs

**DOI:** 10.1016/j.jped.2024.05.003

**Published:** 2024-06-29

**Authors:** Marcia L. Costa, Maria Albertina Santiago Rego, Flavia Cardoso Rodrigues, Sandy S. Pinheiro, Marcela O. Deus, Alexandre S. Moura

**Affiliations:** a Universidade Professor Edson Antônio Velano - UNIFENAS, Programa de Mestrado Ensino em Saúde, Belo Horizonte, MG, Brazil; b Universidade Federal de Minas Gerais, Faculdade de Medicina, Departamento de Pediatria, Belo Horizonte, MG, Brazil; c Hospital Metropolitano Odilon Behrens, Unidade de Cuidados Neonatais, Belo Horizonte, MG, Brazil; d Universidade Professor Edson Antônio Velano - UNIFENAS, Curso de Medicina, Belo Horizonte, MG, Brazil; e Faculdade Santa Casa, Programa de Pós-Graduação em Medicina e Biomedicina, Belo Horizonte, MG, Brazil

**Keywords:** Competency-based medical education, Entrustable professional activities, Medical residency, Pediatrics, Neonatal care, Perinatology

## Abstract

**Objective:**

Define and develop a set of entrustable professional activities (EPAs) to link clinical training and assessment of the hospital components of neonatal care in neonatology medical residency programs.

**Methods:**

An exploratory study was conducted in two phases using a modified Delphi approach. In the first phase, a committee of five neonatology residency program coordinators drafted an initial set of EPAs based on the national matrix of competencies and on EPAs defined by international organizations. In the second phase, a group of neonatal care physicians and medical residents rated the indispensability and clarity of the EPAs and provided comments and suggestions.

**Results:**

Seven EPAs were drafted by the coordinators´ committee (*n* = 5) and used in the content validation process with a group (*n* = 37) of neonatal care physicians and medical residents. In the first Delphi round, all EPAs reached a content validity index (CVI) above 0.8. The coordinators´ committee analyzed comments and suggestions and revised the EPAs. A second Delphi round with the revised EPAs was conducted to validate and all items maintained a CVI above 0.8 for indispensability and clarity.

**Conclusion:**

Seven entrustable professional activities were developed to assess residents in the hospital components of neonatal care medicine. These EPAs might contribute to implementing competency-based neonatology medical residency programs grounded in core professional activities.

## Introduction

Since the beginning of the 21st century, graduate and postgraduate medical training in different countries has been transitioning to competency-based education (CBE) as a response to the concern regarding the quality of medical education and the lack of social accountability and training flexibility.^1^

In Brazil, a core competencies matrix has been proposed for graduate students, and the Ministry of Education has already established the competencies for most medical specialties and areas of expertise, including neonatology.^2^, ^3^, ^4^ However, the transition to a competency-based model is not straightforward, probably because of the difficulty in assessment.^5^ The gaps between a well-designed competency structure and the assessment of clinical practice might contribute to the problem.^6^

In 2005, the concept of entrustable professional activities (EPA) was introduced in medical education to bridge the gap between competence, assessment, and clinical practice.^7^ An EPA is a unit of professional practice, or a profession-specific task, to be entrusted to students once they have demonstrated the integration of essential knowledge and appropriate skills and attitudes. EPAs should be core activities that define the practice of a specialty. While competencies are descriptors of the individual's personal qualities, EPAs describe the tasks that must be performed in the workplace.^8^

The decision to entrust a student to perform a given professional activity depends on the transfer of responsibility between the supervisor and the medical resident, and it is called an entrustment decision.^9^ Entrustment decisions entail subjectivity, and supervisors often make such decisions in uncertainty.^10^ To help reduce the subjectivity of entrustment, Ten Cate et al. defined levels of supervision, which are assigned to those evaluated according to the skills already acquired and demonstrated by them.^6^

A set of EPAs can be used to define the framework of a specialty curriculum.^8^ An EPA-based curriculum has the potential to link clinical training to the daily work of physicians and can be built using the following steps: (i) identification of core EPAs; (ii) description of each EPA (title, specifications, limitations, required knowledge, skills and attitudes, source of information to inform entrustment decision, expected level of supervision, and expiration date); (iii) definition of tools to monitor and record residents ´ performance (e.g. portfolios); (iv) allowing flexibility in the training pathway(6).

A set of EPAs has already been validated for use in Pediatric Cardiac Critical Care in the United States.^11^ In neonatal care, EPAs have already been described by the American Board of Medical Specialties (ABMS) and by the Royal College of Physicians and Surgeons of Canada (RCPSC).^12^^,^^13^ The curriculum created by the ABMS is based on seven EPAs referring to training in pediatrics, with an additional five EPAs specific to neonatology. The RCPSC establishes 24 EPAs necessary for residency training in neonatology, with increasing complexity.

In the present study, the authors aimed to define and develop a set of EPAs that could be used to link clinical training and assessment of neonatal medicine residents.

## Methods

### Study design

A qualitative study was conducted in two phases to develop EPAs for use in assessing neonatology medical residents in the hospital components of neonatal care. The first study phase involved drafting a list of EPAs and the second phase aimed at content validation using the modified Delphi method.

### Participants

The authors invited coordinators from six neonatal medicine residency programs in Belo Horizonte to participate in the first phase of the study, and five agreed to participate. The invited hospitals were referral centers for perinatal health in Minas Gerais State, being responsible for the labor and delivery of high-risk pregnant women and the care of their newborn babies.

The number of participants in the first phase was defined based on a similar study that invited five pediatric intensive care unit (PICU) physicians to draft a set of EPAs for Dutch PICU fellows.^14^ For the second phase, the authors invited a convenient sample of 50 neonatal care physicians and medical residents from Minas Gerais, Brazil. The sample size was chosen to reflect the usual size for Delphi studies between 15 and 60.^15^

### Procedures

**Figure 1:**
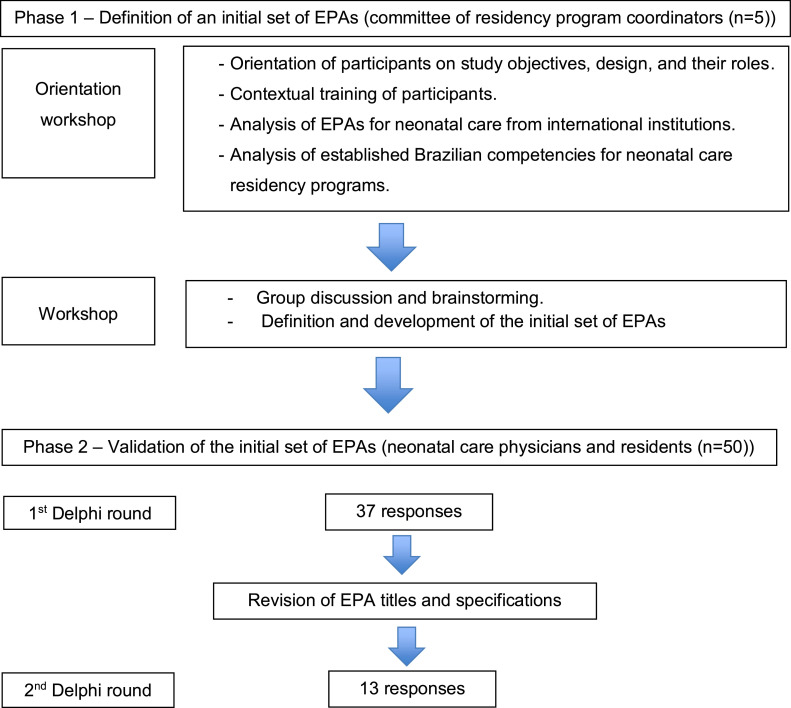
Step-by-step procedure for developing the EPAs framework for Brazilian Neonatal Care Residency Programs.

The stepwise procedure of this study is shown in Figure 1.

### Design of entrustable professional activities (EPAs)

A committee of five neonatal care coordinators drafted a list of EPAs between November 2021 and June 2022. The list was aligned with the competencies related to neonatal hospital care established by the Ministry of Education for Residency Programs of Neonatology in Brazil (2). All committee members had more than ten years of experience in resident supervision in neonatology residency programs.

### Validation of entrustable professional activities using the modified Delphi method

The initial list of EPAs (title, description, and specification) was submitted to a group of neonatal care physicians and residents to obtain consensus using a modified Delphi method.^16^ This method involves the iterative process of developing and distributing a questionnaire of statements, based on the content that needs to be validated, to a group of participants; after each round of responses, the researchers analyze the results and improve the content based on the observations. The process is repeated until the best possible level of consensus is reached.^17^ The Delphi method was chosen because it is an approach to developing consensus widely used in medical education research, particularly in designing EPAs.^9^^,^^17^ Since this method does not require participants to interact directly, it minimizes undue dominance by specific individuals and guarantees anonymity.^17^ The panel members were asked to rate the indispensability and clarity of each EPA on a five-point Likert scale. Finally, panelists were asked to rate the comprehensiveness of the list of EPAs and to provide comments and suggestions for improvement. After each round of consensus, the committee reviewed the results and, if necessary, revised the EPAs. This study phase was conducted between July and September 2022.

### Data analysis

In each round of consensus, the median, mode, interquartile range, and content validity index (CVI) for 'indispensability' and 'clarity' were calculated for the Likert scale data of each EPA. The CVI, the degree to which an instrument has an appropriate sample of items for the measured construct, was calculated as the number of panelists who achieved one of the two highest ratings for each EPA, divided by the total number of panelists.^18^ CVI values ​​can vary from 0 to 1. As a cutoff score, the authors determined that a CVI of 0.8 or greater indicates sufficient content validity, a CVI between 0.70 and 0.79 implies that the item needed revision and a CVI below 0.70 indicates elimination of the item, based on the literature.^19^ If the median for an item was below the predetermined consensus level of 4 for 'indispensability' and 'clarity,' that item was revised by the coordinators´ committee.

Ethical approval was obtained from the Institutional Review Board of Unifenas (CAAE 56,484,122.7.0000.5143), and all participants signed informed consent.

## Results

Five participants from four different medical centers (three public and one private hospital) agreed to participate in the committee responsible for drafting the EPAs. The coordinators´ committee drafted seven EPAs and sent them to a panel of 50 neonatal care physicians and medical residents for content validation.

In the first Delphi round, responses were obtained from 37 (74%) participants. Most respondents were women (89.2%), aged between 40 and 49 years (45.9%), board-certified in neonatology (64.9%), had more than ten years of clinical experience (67.6%), and worked in Belo Horizonte (86.5%). Five (13.5%) panel members were neonatal care residents, and four (10.8%) neonatal care physicians had less than five years of clinical experience.

**Figure 2:**
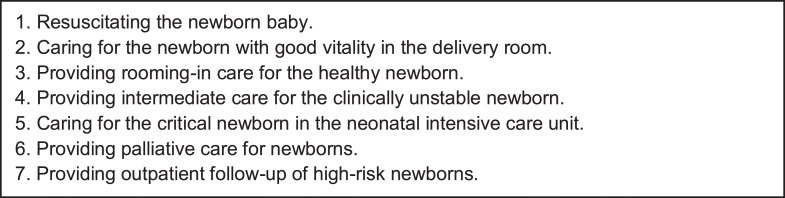
Final list of EPAs to be used to assess residents in neonatal medicine residency programs in Brazil.

After the first round, all EPAs had a CVI above 0.8 and reached the predefined threshold for indispensability and clarity. Regarding the comprehensiveness of the proposed EPAs, no suggestions were made for adding another EPA, and the final list is shown in Figure 2.

The following suggestions were incorporated into the preliminary list of EPAs: (i) In EPA 3 (“Providing rooming-in care for the newborn”), the element “promoting breastfeeding” was added, and (ii) in all other EPAs, the element “clinical documentation” was added.

The researchers then conducted a second round to validate the revised EPAs and 13 responses were obtained. The CVI for indispensability and clarity in the second round was similar to that obtained in the first, with scores ranging from 0.97 to 1.

**Figure 3:**
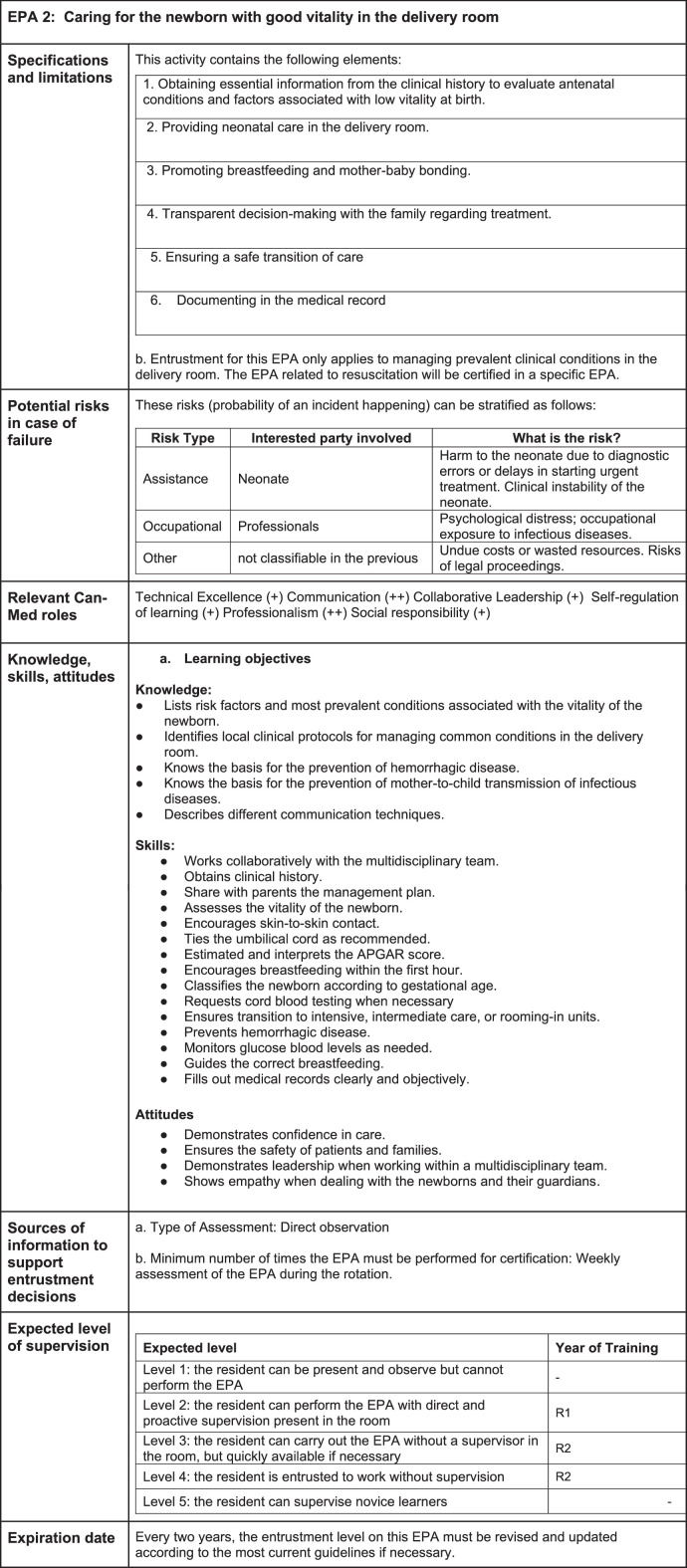
The complete description of EPA 2 - “Caring for the newborn with good vitality in the delivery room.”

A complete description of one of the EPAs is shown as an example (Figure 3). A detailed specification of the seven EPAs is presented as Supplementary Material.

## Discussion

In the present study, the authors reached a consensus on seven EPAs to be used in designing and assessing neonatology residency programs. By defining the EPAs' titles and describing their specifications and limitations, the authors aimed to contribute to the initial steps in implementing EPA-based assessment in neonatology training in Brazil.

Other specialties in Brazil have been discussing and defining EPAs for specialty training. The Brazilian Federation of the Association of Gynecology and Obstetrics (FEBRASGO) has already defined 21 EPAs for residency training.^20^ As far as the authors know, this study is the first to define and develop a set of EPAs as the basis for competency-based teaching of neonatology in Brazil.

An EPA-based curriculum must be constructed by a broad group of stakeholders involved in the specialist's daily routine.^21^ Although our consensus group was composed mostly of individuals with extensive experience, the authors intentionally included medical residents and early career specialists, who comprised almost a quarter of the participants.

In addition, the EPAs must reflect the context where the physician will practice after training. The authors used the Delphi method to reach a consensus on the EPAs that reflect the practice of the neonatal care specialist in Brazil. It is important to emphasize that the objective of the Delphi method is not necessarily to obtain absolute consensus on all items but to achieve the maximum possible convergence of opinion.^15^ The modified Delphi method was chosen because it has been widely used in studies aiming at defining and validating EPAs in medical education. In most of these studies, consensus is reached in two or three rounds, and a high level of agreement between such responses was observed, similar to what was found in the present study.^14^^,^^21^^,^^22^

The number of EPAs that need to be assessed and entrusted in a medical residency program varies according to the area of expertise and regional/national legal requirements for certification. An excessive number of EPAs may risk increasing the complexity of assessment and the demand on clinical supervisors. Conversely, broader and fewer EPAs can reduce the complexity and allow a more holistic view of the resident.^23^ Ten Cate suggests an adequate number of EPAs for a complete residency program should vary between 20 and 30.^6^ However, a recent Dutch study defined only nine EPAs to train Pediatric Intensive Care Unit residents.^15^ In addition, the American Board of Pediatrics (ABP) has proposed ten EPAs for pediatric intensive care medicine and five for neonatology.^12^ Finally, a Delphi study with program directors of the Accreditation Council for Graduate Medical Education (ACGME)-certified neonatal-perinatal medicine fellowships defined thirteen EPAs for neonatology ^22^. In this study, the authors defined seven EPAs, which seemed adequate for a subspecialty in which the resident has already been trained in pediatrics.

When defining the set of EPAs for this study, the authors followed a framework based on “service provision,” one of the most commonly used.^24^ In such a perspective, the EPAs are broad and general, reflecting activities as they are usually assigned to the training physician. Entrustment decisions in this “service provision” framework assume adequate experience with diseases and procedures usually encountered during these services.^24^ A drawback of this framework is its lack of case specificity, which demands careful attention to sampling in assessment and makes it challenging for summative entrustment decisions.^25^

A limitation of the study is the national validity of the set of EPAs, as the original list of EPAs was drafted by experts from a single state in Brazil, and consensus was obtained from specialists from the same region. However, since the list of EPAs was built considering the national matrix of competencies, the authors might assume neonatal medicine residency programs from other parts of Brazil could use it as components of a competency-based assessment strategy.

Seven EPAs were defined and developed for use in neonatal medicine residency programs. Implementing competency-based assessment in postgraduate medical education is challenging and this set of EPAs might be an important step towards operationalizing such strategy in Neonatal Care training.

## Conflicts of interest

The authors declare no conflicts of interest.

## Appendix Supplementary materials

Image, application 1
