# Supplementary material for: Validation of entrustable professional activities for use in neonatal care residency programs
Source: J Pediatr (Rio J). 2024 Jun 29;100(6):627–32. doi: 10.1016/j.jped.2024.05.003 (PMC11662742; doi:10.1016/j.jped.2024.05.003)
Supplement: Supplementary file 1 [file mmc1.docx]

JPED-D-23-00440 – Supplementary Material

**Supplementary material.** Specification of the EPAs that can be used to assess residents in neonatal medicine programs.

| 1. 1. Resuscitating the newborn baby. 2. 2. Caring for the newborn with good vitality in the delivery room. 3. 3. Providing rooming-in care for the healthy newborn. 4. 4. Providing intermediate care for the clinically unstable newborn.   5. Caring for the critical newborn in the neonatal intensive care unit.  6. Providing palliative care for newborns.  7. Providing outpatient follow-up of high-risk newborns. |
| --- |

| **EPA 1: Resuscitating the newborn baby** | |
| --- | --- |
| **Specifications**  **and limitations** | This activity contains the following elements:   \| 1. Recognizing the newborn who needs resuscitation \| \| --- \| \| 2. Leading a multidisciplinary team, before, during and after resuscitation. \| \| 3. Applying the steps of neonatal resuscitation \| \| 4. Performing procedures such as airway management and placement of venous vascular catheters. \| \| 5. Clinically stabilizing the newborn. \| \| 6. Providing safe transportation for the newborn. \| \| 7. Carrying out the transition of care to the appropriate clinical setting. \| \| 8. Documenting in the medical record \|   b. Entrustment for this EPA only applies to resuscitation in the delivery room and neonatal unit. It does not include ongoing management after resuscitation. |

| **EPA 2: Caring for the newborn with good vitality in the delivery room** | |
| --- | --- |
| **Specifications**  **and limitations** | This activity contains the following elements:   \| 1. Obtaining essential information from the clinical history to evaluate antenatal conditions and factors associated with risk for resuscitation at birth. \| \| --- \| \| 2. Providing neonatal care in the delivery room. \| \| 3. Promoting breastfeeding and mother-baby bonding. \| \| 4. Transparent decision-making with the family regarding treatment. \| \| 5. Ensuring a safe transition of care \| \| 6. Documenting in the medical record \|   b. Entrustment for this EPA only applies to managing prevalent clinical conditions in the delivery room. The EPA related to resuscitation will be certified in a specific EPA. |

| **EPA 3: Providing rooming-in care for the healthy newborn.** | |
| --- | --- |
| **Specifications**  **and limitations** | This activity contains the following elements:   \| 1. Obtaining essential information on maternal and neonatal history. \| \| --- \| \| 2. Performing physical examination of the newborn. \| \| 3. Indicating and interpreting neonatal screening tests. \| \| 4. Identifying the main physiological or pathological changes in the newborn. \| \| 5. Promoting breastfeeding. \| \| 6. Indicating treatment for the patient’s clinical condition. \| \| 7. Guiding and supporting the mother and the family in caring for the newborn. \| \| 8. Preparing the discharge plan according to biological conditions and social vulnerability; \| \| 1. Documenting in the medical records. \|   b. Entrustment for this EPA only applies to managing prevalent clinical conditions in this clinical setting (neonatal jaundice, newborn of diabetic mother, hypoglycemia, risk of congenital infections, withdrawal syndrome, low weight gain, microcephaly. |

| **EPA 4: Providing intermediate care for the clinically unstable newborn.** | |
| --- | --- |
| **Specifications**  **and limitations** | This activity contains the following elements:   \| 1. Encouraging the Kangaroo method. \| \| --- \| \| 2. Establishing a multidisciplinary therapeutic plan \| \| 3. Assessing the growth and development of the newborn \| \| 4 .Promoting health and preventing diseases. \| \| 5. Formulating neonatal and post-neonatal prognosis. \| \| 6. Documenting in the medical record \| \| 7. Organizing a multidisciplinary care plan upon hospital discharge \|   b. Entrustment for this EPA only applies to managing prevalent clinical conditions in intermediate care. |

| **EPA 5: Caring for the critical newborn in the neonatal intensive care unit.** | |
| --- | --- |
| **Specifications**  **and limitations** | This activity contains the following elements:   \| 1. Assessing the immediate actions needed to stabilize the newborn \| \| --- \| \| 2. Obtaining essential information from the clinical history \| \| 3. Performing a targeted physical examination \| \| 4 Formulating diagnostic hypotheses \| \| 5 Indicating and evaluating laboratory tests \| \| 6 Establishing a therapeutic plan for the clinical condition of the newborn \| \| 7 Prescribing. \| \| 8. Performing procedures such as inserting an umbilical catheter, orotracheal intubation and lumbar puncture. \| \| 9. Preventing and addressing complications of prematurity. \| \| 10. Sharing decisions with the family in a transparent manner. \| \| 11. Carrying out the transition of care \| \| 12. Documenting in the medical record \|   b. Entrustment for this EPA only applies to managing prevalent clinical conditions in the neonatal care unit (prematurity complications, severe congenital malformation, perinatal asphyxia, neonatal sepsis) |

| **EPA 6: Providing palliative care for newborns.** | |
| --- | --- |
| Specifications  and limitations | This activity contains the following elements:   \| 1. Identify conditions in the prenatal, peripartum and neonatal periods that require palliative care. \| \| --- \| \| 2 Formulating neonatal and post-neonatal prognosis. \| \| 3 Organizing a multidisciplinary neonatal care therapeutic plan jointly with the mother and family. \| \| 4 Managing pain and discomfort. \| \| 5 Breaking bad news. \| \| 6. Documenting in the medical record \|   b. Entrustment for this EPA only applies to neonates admitted to the neonatal care unit or pregnant women in the maternity unit. |

| **EPA 7: Providing outpatient follow-up of high-risk newborns.** | |
| --- | --- |
| **Specifications**  **and limitations** | This activity contains the following elements:   \| 1. Analyzing the therapeutic plan upon hospital discharge. \| \| --- \| \| 2. Assessing the growth and development of at-risk newborns. \| \| 3. Establishing a new multidisciplinary care plan. \| \| 4. Sharing care with primary care \| \| 5. Documenting in the medical record \|   b. Entrustment for this EPA only applies to outpatients from the neonatal care unit. |
